# Environmental Occurrence of Potentially Pathogenic Bacteria in the Equine Anesthetic Induction and Recovery Box: A Single-Center Study

**DOI:** 10.3390/ani16050712

**Published:** 2026-02-25

**Authors:** Riccardo Rinnovati, Federica Meistro, Maria Virginia Ralletti, Paola D’Angelo, Alessandro Spadari, Edoardo Zingariello, Claudia Pollera, Laura Stancampiano

**Affiliations:** 1 Department of Veterinary Medical Sciences, University of Bologna, 40064 Ozzano dell’Emilia, BO, Italy; federica.meistro@unibo.it (F.M.); virginia.ralletti@unibo.it (M.V.R.); laura.stancampiano@unibo.it (L.S.); 2 Burren Veterinary Clinic (BurrenVets), Circular Road, Ennistymon, V95 H6XD Co. Clare, Ireland; 3 Department of Veterinary Medicine and Animal Sciences, University of Milan, Via dell’Università 6, 26900 Lodi, MI, Italy; claudia.pollera@unimi.it

**Keywords:** surgical site infection, equine surgery, environmental contamination, anesthesia recovery box, infection control, opportunistic bacteria

## Abstract

In horses, surgical site infections (SSIs) are multifactorial complications influenced by patient-related, procedural, and environmental factors. While perioperative contamination has been extensively studied in the operating theater, the anesthetic induction and recovery environment has received limited attention. The aim of this study was to characterize bacterial contamination within an equine anesthetic induction and recovery stall and to evaluate its spatial and temporal distribution. Environmental samples were collected from four predefined locations within the stall at three time points: before patient admission, after recovery from general anesthesia, and after routine cleaning and disinfection. Samples were analyzed using standard aerobic culture techniques, and bacterial species were identified. Bacterial contamination was detected at all sampling locations and time points. Contamination increased significantly after recovery from general anesthesia and was most pronounced in areas associated with moisture retention, such as the groove between floor tiles. The bacterial species identified were predominantly opportunistic environmental organisms. No direct association between environmental contamination and surgical site infections could be established. However, the constant presence of potentially pathogenic bacteria indicates that the anesthetic induction and recovery stall represents a relevant component of the perioperative environment. These findings support the inclusion of the recovery area in infection control strategies aimed at reducing perioperative risk in equine surgical patients.

## Simple Summary

Surgical site infections (SSI) are recognized complications in horses undergoing surgery and are influenced by several factors. While most research has focused on the operating theater and the surgical wound itself, less attention has been given to the environment in which horses recover from general anesthesia. This study examines bacterial contamination in the anesthetic induction and recovery stall of an equine surgical facility. Samples were collected from different areas of the stall at various times: before surgery, after recovery from anesthesia, and after cleaning. Bacteria were consistently present in the recovery environment, particularly after the horse had recovered from anesthesia, and were most frequently detected in specific areas of the stall. The bacteria identified were mainly environmental species that can act as opportunistic pathogens. These findings suggest that the recovery stall should be considered as part of infection control strategies in equine hospitals. Understanding when and where bacteria persist in the recovery environment may help improve hygiene practices and reduce the overall risk of infection in horses undergoing surgery.

## 1. Introduction

Postoperative complications remain a relevant clinical concern in equine surgery despite substantial advances in surgical techniques, anesthesia, and perioperative management [1,2]. Among these, surgical site infections (SSIs) are consistently reported as one of the most frequent and clinically significant complications, with important consequences in terms of morbidity, prolonged hospitalization, increased costs, and reduced likelihood of return to athletic performance [3,4,5,6,7]. In horses, SSIs occur across a wide range of surgical procedures, with a particularly high incidence following orthopedic internal fixation and exploratory laparotomy. SSI rates of approximately 14% have been reported after orthopedic internal fixation, with affected horses being significantly less likely to survive to discharge [7]. Following colic surgery, recent retrospective data indicate SSI rates exceeding 25%, with repeated laparotomy and postoperative management factors, including wound management, postoperative abdominal bandaging, antimicrobial administration, and postoperative monitoring, significantly influencing infection risk and outcome [8]. In addition, a recent randomized controlled trial demonstrated that postoperative abdominal bandaging applied during anesthetic recovery after emergency equine laparotomy was associated with a reduced incidence of surgical site infections, underscoring the clinical relevance of the recovery phase in SSI prevention [9]. More broadly, postoperative morbidity following general anesthesia remains a significant concern in adult horses, with systematic evidence showing that overall postoperative complications, including surgical site complications, occur in a substantial proportion of cases and are markedly more prevalent after colic surgery compared with elective procedures, underscoring the clinical relevance of peri-recovery factors in SSI risk [10].

The pathogenesis of SSIs is multifactorial, involving interactions between patient-related factors, surgical variables, and perioperative microbial exposure [3]. Increasing concern regarding antimicrobial resistance has further emphasized the importance of perioperative infection control strategies, including environmental hygiene and antimicrobial stewardship [2,11]. In this context, a broader evaluation of the perioperative environment is increasingly advocated, beyond patient- and procedure-related factors alone [12,13].

In addition to acute musculoskeletal trauma and cardiopulmonary complications, SSIs may also be associated with poor recovery quality, potentially as a result of mechanical disruption of protective coverings and increased exposure of surgical wounds to contaminated environmental surfaces during anesthetic recovery [4,14]. Experimental and clinical observations have also linked disruption of protective drapes during recovery to postoperative skin contamination and increased likelihood of incisional drainage [15]. Recovery quality has been shown to be influenced by identifiable pre- and intraoperative predictors (e.g., age, breed, emergency status, anesthesia/recovery duration, and intraoperative hypoxemia/hypotension). These factors may modulate the duration and intensity of patient–environment interaction during recovery, supporting the relevance of this phase when considering perioperative environmental exposure [16,17].

Although intraoperative contamination has been widely investigated, the potential role of the anesthetic induction and recovery stall as a source of environmental bacterial exposure has not been specifically addressed. Recent bacteriological surveys confirm that contamination of surgical sites may occur despite standard aseptic measures, supporting the hypothesis that environmental and temporal factors may modulate SSI development [18]. In parallel, non-antibiotic preventive approaches have been explored with promising preliminary results in equine laparotomy patients [19]. Therefore, this prospective study aimed to characterize the environmental occurrence and spatial–temporal distribution of potentially pathogenic bacteria within the anesthetic induction and recovery stall of an equine surgical unit to address its role within perioperative infection risk.

## 2. Materials and Methods

### 2.1. Study Design and Setting

This study was conducted as a prospective observational study aimed at describing the environmental occurrence and spatial–temporal distribution of potentially pathogenic bacteria within an equine anesthetic induction and recovery box. Environmental sampling was carried out over a 12-month period from February 2019 to February 2020 at an equine university teaching hospital during routine clinical activity. The study was intentionally designed as descriptive and exploratory, with the primary objective of characterizing environmental bacterial contamination patterns. It was not designed to investigate clinical outcomes, including the incidence or causation of surgical site infections, and no changes to standard clinical or hygiene protocols were introduced for research purposes. During the study period, the equine surgical unit routinely performed surgical procedures as part of regular clinical activity. Environmental sampling was not carried out for every procedure, but only for those in which the complete sampling protocol could be applied in a standardized manner. Accordingly, only procedures with a full set of environmental samples collected at all three time points were included in the final analysis. Postoperative monitoring for surgical site infection was limited to the in-hospital period following anesthesia and surgery. The duration of hospitalization varied according to the type of surgical procedure. Horses undergoing exploratory laparotomy and urogenital surgery were typically hospitalized for up to 7 days postoperatively, whereas horses undergoing orthopedic procedures were hospitalized for a maximum of 3 days. No systematic post-discharge follow-up was performed as part of the study.

### 2.2. Anesthetic Induction and Recovery Box

Anesthetic induction and recovery from general anesthesia were routinely performed in the same enclosed recovery box. The box consisted of a rubber-tiled floor with grooves between tiles, padded walls, and floor–wall junctions. This flooring system is routinely used at the study institution and is designed to provide traction and cushioning while allowing effective cleaning and disinfection. The recovery box was used repeatedly for horses undergoing different categories of surgical procedures throughout the study period, resulting in regular exposure to biological material, moisture, and mechanical contact. No structural modifications or experimental interventions were applied to the box, allowing environmental contamination to be assessed under real-world clinical conditions.

### 2.3. Sampling Method

**Figure 1:**
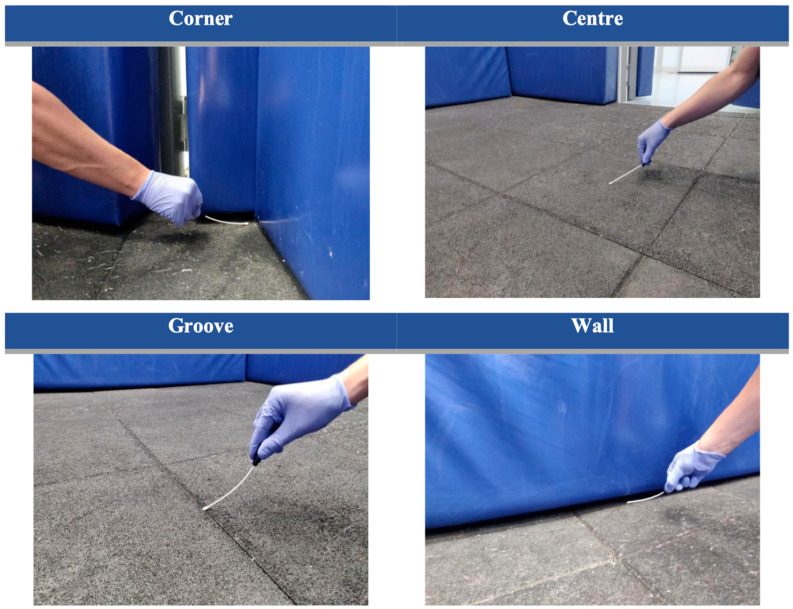
**Environmental sampling locations within the equine anesthetic induction and recovery box.** Sterile swabs were used to collect samples from four predefined areas: the corner of the stall (Corner), the central floor area (Center), the groove between floor tiles (Groove), and the junction between the floor and the wall (Wall). These locations were selected to represent areas with differing surface characteristics, levels of contact, and propensity for moisture retention. These locations were deliberately chosen to represent areas differing in surface morphology, likelihood of moisture retention, and accessibility during routine cleaning and disinfection, thereby allowing for exploration of potential spatial differences in environmental contamination.

Environmental samples were collected using sterile cotton swabs moistened with sterile saline solution. Sampling was performed by trained operators wearing clean, powder-free gloves. Each swab was applied to the sampled surface using even pressure, moving consistently from left to right while keeping the swab flat against the surface. Each sampling area measured approximately 10 × 10 cm in order to standardize surface coverage across all samples. Four predefined locations within the anesthetic induction and recovery box were selected for sampling: (i) the corner of the box, (ii) the center of the box, (iii) the groove between floor tiles, and (iv) the junction between the floor and the wall (Figure 1).

### 2.4. Sampling Time Points

Environmental sampling was performed at three standardized time points during each surgical case: T1: before patient admission into the recovery box; T2: immediately after recovery from general anesthesia; T3: after completion of routine cleaning and disinfection procedures. At each time point, all four predefined locations were sampled, resulting in a total of 12 environmental samples per surgical procedure.

### 2.5. Management of Patients During Preparation and Anesthetic Recovery

All horses included in the study underwent routine perioperative preparation in accordance with standard institutional clinical protocols routinely applied at the university equine hospital. This preparation included brushing the coat and cleaning the hooves; in orthopedic cases, shoes were removed before surgery as part of standard clinical practice. Following the completion of the surgical procedure, horses were transferred to the anesthetic induction and recovery box and positioned in lateral recumbency in the central area of the box. Recovery from general anesthesia was unassisted in all cases, allowing environmental exposure during recovery to be assessed under standard operating conditions. Surgical site coverage during anesthetic recovery followed routine clinical practice at the study institution and varied according to the type of surgical procedure. Horses undergoing orthopedic procedures had surgical sites routinely bandaged during recovery. In contrast, horses undergoing exploratory laparotomy did not receive abdominal bandaging while in the anesthetic recovery box; abdominal bandages were applied only after the horse had exited the recovery stall. No additional protective measures, experimental interventions, or modifications to routine recovery management were introduced for the purposes of the study.

### 2.6. Cleaning and Disinfection Procedures

Cleaning and disinfection of the anesthetic induction and recovery box were performed by one of two authorized operators following a standardized institutional protocol. The procedure consisted of an initial mechanical washing phase using water under pressure to remove visible organic material, without the use of additional surfactant detergents, followed by application of a potassium peroxymonosulfate-based disinfectant (Virkon™ S, Lanxess, Köln, Germany) at a 5% concentration. The disinfectant solution was applied to all surfaces and left in contact for approximately 30 min, in accordance with the manufacturer’s recommendations. After the contact period, surfaces were rinsed with water, and post-disinfection environmental sampling (T3) was performed after completion of the drying phase; drying time was not standardized or formally recorded.

### 2.7. Microbiological Processing

Immediately after collection, all swabs were placed in Amies transport medium (Thermo Scientific^TM^, Amies Agar Gel Transport Swab, Thermo Fisher, Rodano (MI), Italy), labeled, and stored at 4 °C, and were submitted within 24 h to the microbiology laboratory of the Department of Veterinary Medicine and Animal Science, University of Milan, for bacteriological analysis. Samples were streaked onto 5% sheep blood agar plates and incubated under aerobic conditions at 37 ± 2 °C for 24–48 h. Following incubation, bacterial growth was assessed based on macroscopic colony characteristics, including size, morphology, pigmentation, and hemolysis. Microscopic evaluation included Gram staining and examination under light microscopy at 40× and 100× magnification. Plates showing no bacterial growth were classified as sterile. Plates displaying light, mixed growth were classified as negative, as they were considered indicative of low-level, non-specific environmental contamination and were not suitable for reliable bacterial identification. Positive cultures were defined as plates showing pure growth (classified as light, moderate, or heavy) or mixed growth involving up to two bacterial genera, allowing species-level identification. Plates showing mixed growth involving more than two bacterial genera were considered non-interpretable and were excluded from species-level analyses.

Bacterial identification was performed using Gram staining, conventional biochemical tests (oxidase and catalase tests), and the API identification system (bioMérieux, Craponne, France), following the manufacturer’s instructions.

Antimicrobial susceptibility testing was not performed, as the scope of the study was limited to environmental characterization rather than the assessment of clinical pathogenicity or resistance profiles.

### 2.8. Data Organization

Environmental data were organized prior to statistical analysis in order to facilitate the evaluation of spatial and temporal patterns of bacterial contamination within the anesthetic induction and recovery box. Each environmental sample was classified according to sampling location (corner, center, groove between floor tiles, and floor–wall junction) and sampling time point (before patient admission into the recovery box, after recovery from general anesthesia, and after cleaning and disinfection). Bacterial species isolated from positive cultures were recorded and classified according to genus and species. Isolates were subsequently ordered according to descending frequency of isolation to identify the most frequently occurring potentially pathogenic bacteria within the sampled environment. Surgical procedures were categorized as coeliotomy, orthopedic surgery, or urogenital surgery. In addition, the operator responsible for cleaning and disinfection of the recovery box was recorded for each sampling session.

### 2.9. Statistical Analysis

Statistical analysis was performed to evaluate spatial and temporal patterns of environmental contamination within the anesthetic induction and recovery box. Descriptive statistics were first applied. Data were aggregated by sampling time point and by sampling location. For each sampling time point, the mean number of positive samples per surgical procedure was calculated based on four samples per procedure; similarly, for each sampling location, the mean number of positive samples per surgical procedure was calculated based on three samples per location. Percentages of positive samples were calculated in parallel to facilitate comparison across locations, time points, types of surgical procedures, and cleaning operators. Following descriptive analysis, inferential statistical tests were applied. The Wilcoxon matched-pairs signed-rank test was used to compare contamination between sampling time points and between sampled locations within the same surgical procedure. The Kruskal–Wallis test was used to evaluate differences in contamination between categories of surgical procedures and between the two operators responsible for cleaning and disinfection. Statistical analyses were performed with an exploratory intent to describe spatial and temporal patterns of environmental contamination rather than to test predefined hypotheses. Therefore, *p*-values are presented descriptively and were not adjusted for multiple comparisons. All statistical analyses were performed using Stata version 18 (StataCorp, College Station, TX, USA). Statistical significance was set at *p* < 0.05.

## 3. Results

### 3.1. Study Population and Sampling Overview

Nineteen surgical procedures were included in the study between February 2019 and February 2020. Horses ranged in age from <1 to 19 years (mean ± SD: 8.9 ± 6.9 years). Surgical procedures comprised three celiotomies, nine orthopedic procedures, and seven urogenital surgeries. No cases of surgical site infection were diagnosed during the study period. The distribution of surgical procedures included in the study reflected routine clinical activity during the study period and was not intentionally predefined. None of the 19 horses developed clinical signs of surgical site infection during the postoperative hospitalization period. A total of 12 samples per procedure and 228 environmental samples were analyzed.

### 3.2. Overall Culture Results

Of the 228 environmental samples, 36 samples (15.8%) showed no bacterial growth and were classified as sterile, while 15 samples (6.6%) showed light mixed growth and were classified as negative. The remaining 177 samples (77.6%) yielded positive bacterial cultures. Positive cultures were detected at each sampling location and each sampling time point, indicating widespread environmental bacterial contamination of the anesthetic induction and recovery box throughout the study period.

### 3.3. Spatial Distribution of Positive Samples

Three samples were collected from each predefined area for each surgical procedure, resulting in 57 samples per area. When results were grouped according to sampling location, the groove between floor tiles showed the highest proportion of positive cultures (92.8%), followed by the center of the stall (82.5%) and the floor–wall junction (77.2%). The corner was the least contaminated area, although in this site as well, 59.7% of the samples remained culture-positive (Table 1). Detailed distributions of sterile and negative samples according to sampling location and time point are provided in the Supplementary Materials (Supplementary Tables S1 and S2). Individual contamination patterns across sampling locations are illustrated in Supplementary Figure S1. Additional descriptive data on positive samples are provided in the Supplementary Materials (Tables S3 and S4). Pairwise comparisons demonstrated that positive cultures were significantly more likely to be obtained from the center than from the corner (*p* = 0.0331). The groove between tiles was significantly more contaminated than the center (*p* = 0.0143) and the wall (*p* = 0.0493). No significant difference was found between the center and the wall (*p* = 0.4161). Both the groove (*p* = 0.0017) and the wall (*p* = 0.047) were significantly more likely to yield positive cultures than the corner.

### 3.4. Temporal Distribution of Positive Samples

Four environmental samples were collected at each sampling time point for each surgical procedure, resulting in a total of 76 samples per time point. When samples were analyzed according to sampling time, the highest proportion of positive cultures was observed after recovery from general anesthesia (T2), with 88.2% of samples testing positive. In comparison, 75.0% of samples were positive before patient admission into the induction/recovery stall (T1), while 68.4% were positive after cleaning and disinfection (T3) (Table 2). Additional descriptive data on positive samples are provided in the Supplementary Materials (Tables S3, S4, S6, and S7). Individual contamination trends across sampling time points are shown in Supplementary Figure S2. Using the Wilcoxon matched-pairs signed-rank test, samples collected at T2 were significantly more likely to be culture-positive than those collected at T1 (*p* = 0.0345) and T3 (*p* = 0.006). No statistically significant difference was detected between T1 and T3.

### 3.5. Bacterial Species Identified

A wide range of bacterial species was isolated from positive environmental samples. Mixed bacterial growth was the most frequent finding, observed in 41 samples. Among single-species isolates, Gram-negative environmental opportunistic bacteria predominated, particularly species commonly associated with moist environments. The most frequently isolated organisms were *Pseudomonas fluorescens* (19/177 culture-positive samples), *Alcaligenes faecalis* (17/177 culture-positive samples), *Pseudomonas putida* and *Aeromonas hydrophila* (14/177 culture-positive samples), and *Burkholderia cepacian* (12/177 culture-positive samples). Other recurrent isolates included *Ralstonia picketti* (9/177 culture-positive samples); Pseudomonas *alcaligenes*, *Ochrobactrum anthropi*, and *Moraxella* spp. (7/177 culture-positive samples); *Comamonas testosteroni* (6/177 culture-positive samples); and *Moraxella* spp. (7/177 each). Less frequently isolated species included *Pseudomonas stutzeri* (4/177 culture-positive samples); *Bacillus* spp., *Pseudomonas oryzihabitans*, *Rhizobium radiobacter*, and *Photobacterium damselae* (3/177 culture-positive samples); *Cupriavidus pauculus*, *Citrobacter freundii*, *Achromobacter xylosoxidans*, and *Pseudomonas luteola* (2/177 each); and *Corynebacterium pseudodiphthericum*, *Shewanella putrefaciens* group, and *Aerococcus viridans* (1/177 each). No obligate equine commensals or host-specific pathogens were identified in any environmental samples. The complete list of bacterial species isolated from environmental samples and their frequency of detection is provided in the Supplementary Materials (Table S5).

### 3.6. Effect of Cleaning Operator and Type of Surgery

Environmental contamination was further analyzed according to the operator responsible for cleaning and disinfection of the induction/recovery stall. Operator 1 performed cleaning and disinfection procedures on 12/19 sampling sessions (63.2%), while operator 2 did so on 7/19 sessions (36.8%). Results by cleaning operator are illustrated in Supplementary Figures S3 and S4. Operator-specific results are reported in the Supplementary Materials (Tables S8–S12). Although numerical differences in the mean number of positive samples were observed between operators at T2 and T3, no statistically significant differences were detected either in contamination levels after disinfection (T3) (*p* = 0.4267) or in the reduction in contamination between T2 and T3 (*p* = 0.1945), as assessed using the Kruskal–Wallis test.

### 3.7. Results Grouped by Type of Surgical Procedure

**Figure 2:**
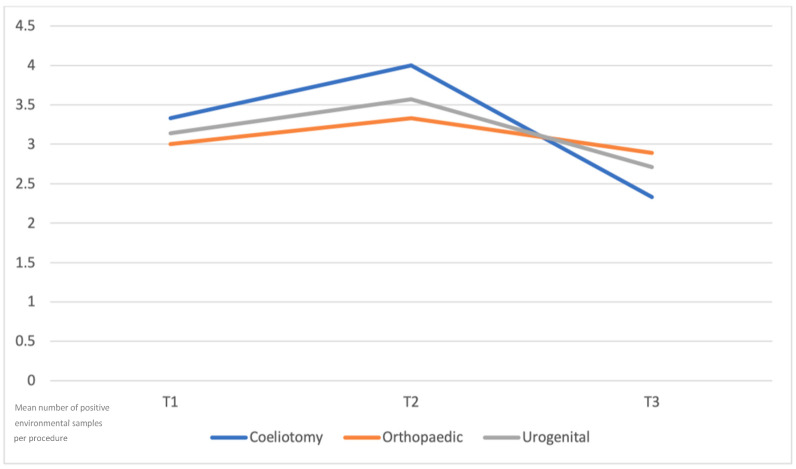
**Mean number of positive environmental samples across sampling time points (T1–T3) according to the type of surgical procedure**. Values represent descriptive means calculated per procedure; no measures of variability are shown. No statistically significant differences were detected among surgical categories; the figure illustrates the shared temporal pattern of environmental contamination.

Surgical procedures included celiotomies (3/19 procedures, 15.8%), orthopedic surgeries (9/19 procedures, 47.4%), and urogenital surgeries (7/19 procedures, 36.8%). When results were grouped according to the type of surgical procedure, no statistically significant differences in environmental contamination were identified among celiotomies, orthopedic procedures, and urogenital surgeries (*p* = 0.3740). However, a similar temporal pattern was observed across all surgical categories, with an increase in positive samples after recovery from general anesthesia (T2), followed by a decrease after cleaning and disinfection (T3) (Figure 2). Descriptive analysis showed comparable patterns of temporal and spatial contamination across all three categories of surgical procedures. Detailed distributions of positive samples according to area and time for each surgical category are provided in the Supplementary Materials (Tables S12–S17).

## 4. Discussion

Despite the extensive literature investigating bacterial contamination within the operating theater [12,20,21,22], the surgical site [3,8,9,18,23], and the operating team [12,21,24,25], the anesthetic induction and recovery stall has received limited attention as a potential environmental contributor to perioperative microbial exposure in horses. Most available studies have focused on intraoperative contamination or surgical wound management [9,19,26], whereas the recovery phase has remained comparatively underexplored. To the authors’ knowledge, this is one of the first studies to investigate the recovery stall in relation to factors potentially relevant to surgical site exposure during the perioperative period. By focusing on an underexplored component of the perioperative environment, this study expands current understanding of potential environmental contributors to surgical site exposure beyond the operating theater and surgical field.

All bacterial species isolated in this study were classified as emerging opportunistic environmental microorganisms, and no obligate equine or human commensal pathogens were identified. In addition, no horses developed SSIs. These findings are consistent with previous reports indicating that the presence of bacteria in the perioperative environment or even at the surgical site does not necessarily translate into clinical infection [18,27]. It should be acknowledged, however, that equine SSIs may develop several days to weeks after surgery [9], and the present study was not designed to include long-term post-discharge follow-up. As such, the absence of SSI in this cohort should be interpreted within the context of short-term clinical observation rather than as evidence of the lack of clinical relevance of environmental contamination, and delayed-onset surgical site infections, particularly after orthopedic procedures, cannot be excluded [6,7,8]. This distinction between environmental contamination and clinical SSI is well recognized in infection control literature [28], where surface contamination is increasingly regarded as a risk-modifying factor that may contribute to infection development in the presence of additional predisposing conditions, rather than as a direct causal determinant. The absence of SSIs in the present cohort further supports the value of replicating this study in clinical settings with a known or higher incidence of SSIs in order to better explore the potential relationship between environmental contamination patterns and clinically relevant outcomes.

The spatial distribution of bacterial contamination observed in the recovery stall appears biologically and environmentally plausible. Many of the most frequently isolated species, including *Pseudomonas putida*, *Aeromonas hydrophila*, *Ralstonia pickettii*, and *Sphingomonas paucimobilis*, are known to persist in water-associated or moist environments [29,30,31,32,33,34,35,36,37,38,39]. The significantly higher contamination rate observed in the groove between floor tiles (92.8%) is therefore more likely attributable to prolonged moisture retention and reduced drying efficiency than to inadequate cleaning practices. Structurally complex or discontinuous surfaces have been repeatedly identified as persistent environmental reservoirs in healthcare settings [40]. However, in equine anesthetic recovery areas, nonsmooth surfaces are intentionally used to provide traction and reduce the risk of musculoskeletal injury during recovery. This represents an inherent trade-off between patient safety and infection control. Rather than representing a design flaw, structured flooring in recovery stalls should therefore be considered a necessary compromise, requiring optimized cleaning and disinfection protocols to mitigate residual environmental contamination potentially relevant to surgical site exposure [28,40].

From a temporal perspective, environmental contamination increased significantly after recovery from general anesthesia compared with both pre-anesthetic and post-disinfection sampling. This finding highlights the recovery phase as a critical window for environmental contamination. Recovery from general anesthesia in horses is associated with increased movement, contact with the ground surface, and potential disruption of protective dressings, all of which may facilitate bacterial dispersion within the stall [4,10,14,17,41]. The consistent increase in contamination observed at this time point supports the concept of the recovery stall as a dynamic environment in which bacterial load fluctuates in response to patient presence and activity rather than remaining static. Several potential sources of bacterial contamination within the recovery stall must be considered, including the patient, personnel, cleaning equipment, and residual environmental flora. Regarding the patient, no bacteria typically associated with the equine upper respiratory tract or skin microbiota, such as *Streptococcus* spp., were isolated [42,43], suggesting that preoperative preparation procedures were effective in limiting gross contamination. Although the equine cutaneous microbiota has been described [44,45,46], currently available data do not allow for definitive attribution of the environmental bacterial species isolated in this study to either cutaneous or environmental sources, particularly in the context of perioperative exposure. The significantly higher number of positive cultures obtained after recovery (T2) compared with baseline sampling (T1) nonetheless supports the hypothesis that the animal itself may contribute substantially to environmental contamination during this phase. The use of a single stall for both anesthetic induction and recovery may further amplify this effect, as contamination introduced during recovery may persist and increase the baseline environmental bacterial load.

Coagulase-negative staphylococci, which may be associated with both human and equine cutaneous microbiota [47], were not identified in environmental samples. However, based on environmental sampling alone, it is not possible to determine the relative contribution of personnel, animals, or other environmental sources to the observed bacterial contamination [42]. Therefore, while transient contamination related to human activity cannot be excluded, the findings do not allow definitive conclusions regarding the role of personnel in environmental bacterial load.

Across all sampling time points, corners showed the lowest proportion of culture-positive samples (59.7%), followed by wall–floor junctions (77.2%). These percentages represent the proportion of positive environmental samples out of all samples collected at each location and do not reflect a reduction rate or a benchmark of decontamination efficacy. This observation reinforces the concept that environmental design and surface characteristics may represent limiting factors in infection control, independent of operator performance [48].

The present findings should therefore be interpreted descriptively, within the context of environmental exposure potentially relevant to SSI.

From a preventive perspective, when feasible, the use of continuous, non-porous flooring materials instead of tiled surfaces, as well as the physical separation of anesthetic induction and recovery areas, may help reduce environmental bacterial persistence and should be considered within surgical site infection prevention strategies.

The present study has several limitations that should be acknowledged. The sample size was limited and derived from a single center, which may restrict the generalizability of the findings, particularly with respect to high-risk procedures such as celiotomy. Environmental sampling was qualitative and confined to the recovery stall, without parallel sampling of surgical wounds, patients, or personnel, preventing definitive attribution of bacterial sources or transmission pathways. An additional limitation of the present study is that bacterial identification was performed using conventional culture-based and biochemical methods rather than matrix-assisted laser desorption/ionization time-of-flight mass spectrometry (MALDI-TOF). MALDI-TOF has become increasingly established as a reference technique for bacterial identification in veterinary microbiology [49,50]; however, this technology was not available at the institution at the time of sample collection (2019–2020). The methods applied, therefore, reflected standard diagnostic practice at that time and were considered appropriate for the descriptive and environmental scope of the study. Moreover, postoperative follow-up was limited to the hospitalization period, and delayed-onset surgical site infections could not be assessed. Finally, the use of a single stall for both anesthetic induction and recovery may have influenced baseline environmental contamination levels. Future studies should therefore include larger, multicenter populations, incorporate quantitative microbiological sampling, and focus on high-risk surgical procedures with extended post-discharge follow-up to better clarify the clinical relevance of environmental contamination during recovery.

## 5. Conclusions

This study demonstrates that the equine anesthetic induction and recovery stall is consistently contaminated by potentially pathogenic bacteria, with distinct spatial and temporal patterns. Environmental contamination increased after recovery from general anesthesia (T2) and was most pronounced in areas prone to moisture retention, indicating that the recovery environment represents a biologically possible source of perioperative bacterial exposure.

In the context of the multifactorial etiology of SSIs, the constant presence of potentially pathogenic environmental bacteria suggests that the anesthetic induction and recovery stall should be considered among the potential contributors to perioperative risk and a possible weak point within the infection control chain.

Future studies should include larger and multicenter populations, place particular emphasis on high-risk procedures, and incorporate extended post-discharge follow-up and longitudinal sampling of surgical wounds to better clarify the clinical relevance of environmental contamination during the recovery phase.

## Figures and Tables

**Table 1:** **Distribution of positive environmental samples according to sampling location within the anesthetic induction and recovery box**. Data are expressed as the mean number of positive samples per procedure (minimum–maximum) and as the percentage of positive samples for each sampling location.

Sampling Location	Positive Samples per Procedure, Mean (min–max)	Percentage of Positive Samples (%)
Corner	1.79 (1–3)	59.7
Centre	2.47 (0–3)	82.5
Groove	2.79 (1–3)	92.8
Wall	2.32 (0–3)	77.2

**Table 2:** **Percentage of positive environmental samples according to sampling location and sampling time point within the anesthetic induction and recovery box**. Data are expressed as percentages of positive samples for each sampling location (corner, center, groove between floor tiles, and floor–wall junction) at each sampling time point (before patient admission, after recovery from general anesthesia, and after cleaning and disinfection).

Sampling Time	Corner (%)	Centre (%)	Groove (%)	Wall (%)
T1	57.9	84.2	100.0	68.5
T2	64.0	94.7	94.7	94.7
T3	52.6	68.4	84.2	68.4

## Data Availability

The data supporting the findings of this study are not publicly available due to privacy and institutional restrictions but may be provided by the corresponding author upon reasonable request.

## Supplementary Materials

The following supporting information can be downloaded at https://www.mdpi.com/article/10.3390/ani16050712/s1. Table S1: Sterile environmental samples classified according to sampling location and sampling time point; Table S2: Negative environmental samples classified according to sampling location and sampling time point; Figure S1: Stacked line graph illustrating the relationship between sampling location and contamination, expressed as the number of positive environmental samples; Table S3: Positive environmental samples classified according to sampling location and sampling time point; Table S4: Positive environmental samples classified according to sampling location and sampling time point, expressed as the mean number of positive samples per procedure (minimum–maximum); Figure S2: Stacked line graph illustrating the relationship between time of sampling (x-axis) and contamination, expressed as the number of positive samples (y-axi). Table S5: List of bacterial species isolated from positive environmental samples and their frequency of detection; Table S6: Mean number of positive environmental samples per procedure according to sampling time point (T1–T3); Table S7: Percentage of positive environmental samples according to sampling time point (T1–T3); Figure S3: Line graph illustrating the relationship between time of sampling (x-axis) and contamination, expressed as the mean number of positive samples (y-axis) for each of the two operators evaluated; Table S8: Positive environmental samples per procedure for Operator 1, grouped according to sampling time point; Table S9: Positive environmental samples per procedure for Operator 2, grouped according to sampling time point; Figure S4: Column graph illustrating the relationship between the area sampled (x-axis) and contamination, expressed as the mean number of positive samples (y-axis), for each of the two operators evaluated; Table S10: Positive environmental samples per procedure for Operator 1, grouped according to sampling location; Table S11: Positive environmental samples per procedure for Operator 2, grouped according to sampling location; Table S12: Distribution of positive environmental samples from celiotomy procedures according to sampling location and sampling time point; Table S13: Positive environmental samples per procedure from celiotomy procedures, grouped according to sampling time point; Table S14: Positive environmental samples per procedure from celiotomy procedures, grouped according to sampling location; Table S15: Distribution of positive environmental samples from orthopedic procedures according to sampling location and sampling time point; Table S16: Positive environmental samples per procedure from orthopedic procedures, grouped according to sampling time point; Table S17: Positive environmental samples per procedure from orthopedic procedures, grouped according to sampling location; Table S18: Distribution of positive environmental samples from urogenital procedures according to sampling location and sampling time point.
