# Supplementary material for: Environmental Occurrence of Potentially Pathogenic Bacteria in the Equine Anesthetic Induction and Recovery Box: A Single-Center Study
Source: Animals (Basel). 2026 Feb 25;16(5):712. doi: 10.3390/ani16050712 (PMC12983972; doi:10.3390/ani16050712)
Supplement: Supplementary file 1 [file animals-16-00712-s001.zip › animals-4101102-supplementary.pdf]

Supplementary Materials

	Corner	Centre	Groove	Wall
T1	7	2	0	6
T2	4	0	1	1
T3	5	3	2	5

Table S1: Sterile environmental samples classified according to sampling location and sampling time point.

	Corner	Centre	Groove	Wall
T1	1	1	0	1
T2	2	1	0	0
T3	4	3	1	1

Table S2: Negative environmental samples classified according to sampling location and sampling time point

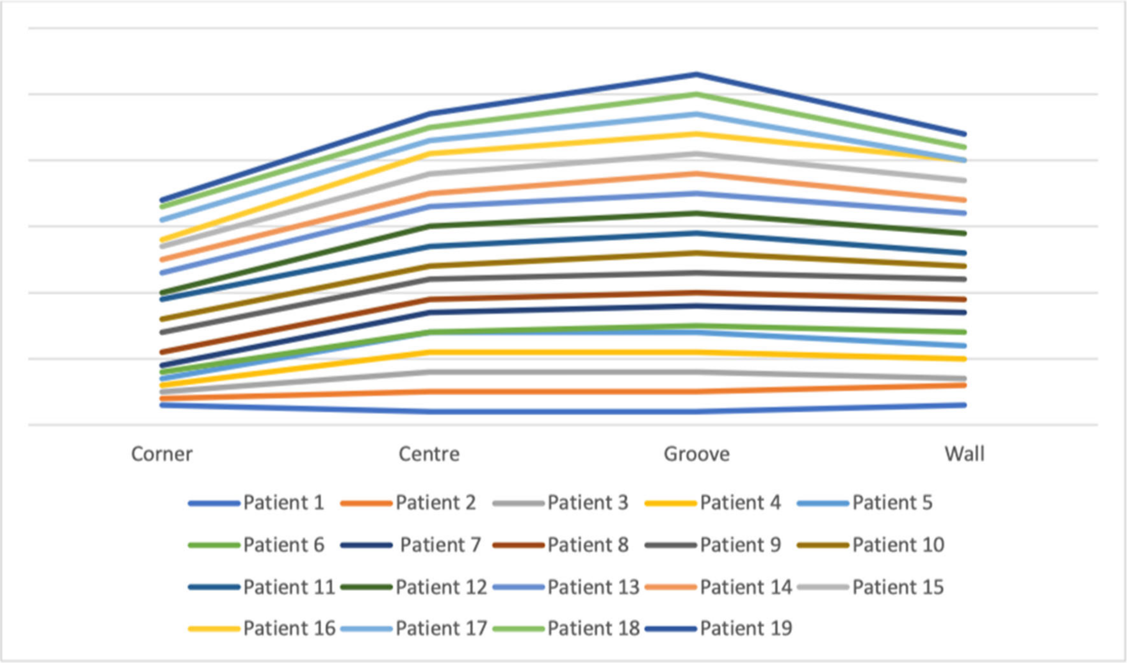

Figure S1: Stacked line graph illustrating the relationship between area sampled (x-axis) and contamination, expressed as the number of positive samples.

	Corner	Centre	Groove	Wall
**T1**	11	16	19	12
**T2**	13	18	18	18
**T3**	10	13	16	13

Table S3: Positive environmental samples classified according to sampling location and sampling time point.

	Positive samples per operation: mean (min-max)	Percentage of positive samples
**T1**	3.11 (2 - 4)	75.0
**T2**	3.53 (1 – 4)	88.2
**T3**	2.74 (0 – 4)	68.4

Table S4: Positive environmental samples classified according to sampling location and sampling time point, expressed as the mean number of positive samples per operation (minimum–maximum).

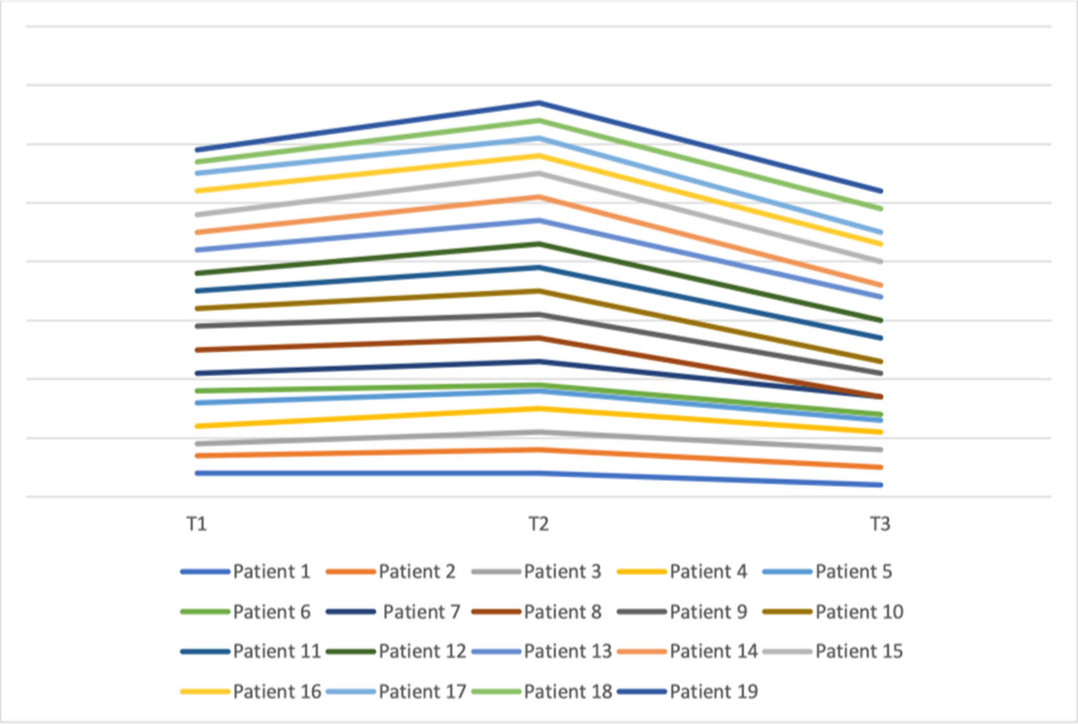

Figure S2: Stacked line graph illustrating the relationship between time of sampling (x-axis) and contamination, expressed as the number of positive samples (y-axis).

Mixed cultures	forty-one samples
Pseudomonas fluorescens	nineteen samples
Alcaligenes faecalis	seventeen samples
Pseudomonas putida and *Aeromonas hydrophila*	fourteen samples each
Burkholderia cepacia	twelve samples
Ralstonia picketti	nine samples
Pseudomonas alcaligenes, Ochrobactrum anthropi and Moraxella spp.	seven samples
Comamonas testosteroni	six samples
Sphingomonas paucimobilis	five samples
Pseudomonas stutzeri	four samples
Bacillus spp., *Pseudomonas oryzihabitans*, *Rhizobium radiobacter*, *Photobacterium damselae*	three samples each
Cupriavidus pauculus, *Citrobacter freundii*, *Achromobacter xyloxidans*, *Pseudomonas luteola*	two samples each
Corynebacterium pseudodiphtericum, *Shewanella putrefaciens* group, *Aerococcus viridans*	one sample each

Table S5: List of bacterial species isolated from positive environmental samples and their frequency of detection.

Operator 1: Positive samples per operation.			
	Mean	Standard deviation	Min-max
T2	3.42	0.9000	1 - 4
T3	2.92	0.9960	1 - 4

Table S6: Mean number of positive environmental samples per operation according to sampling time point (T1–T3).

Operator 2: Positive samples per operation.			
	Mean	Standard deviation	Min-max
T2	3.71	0.49	3 - 4
T3	2.43	1.27	0 - 4

Table S7: Percentage of positive environmental samples according to sampling time point (T1–T3).

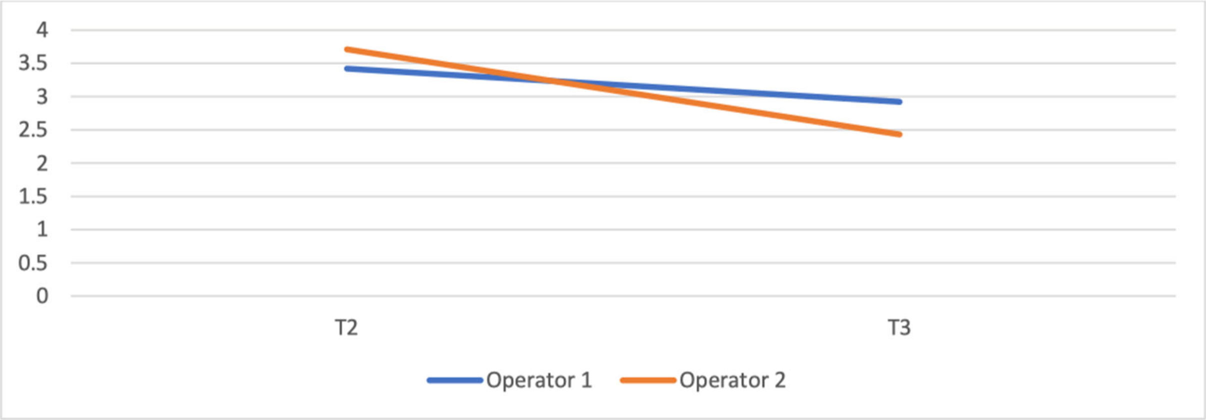

Figure S3: Line graph illustrating the relationship between time of sampling (x-axis) and contamination, expressed as the mean number of positive samples (y-axis) for each of the two operators evaluated.

Operator 1: Positive samples per operation.			
	Mean	Standard Deviation	Min-max
Corner	1.92	0.90	1 – 3
Centre	2.33	0.89	0 – 3
Groove	2.75	0.62	1 – 3
Wall	2.33	0.89	0 - 3

Table S8: Positive environmental samples per operation for Operator 1, grouped according to sampling time point.

Operator 2: Positive samples per operation.			
	**Mean**	**Standard Deviation**	**Min-max**
**Corner**	1.57	0.79	1 – 3
**Centre**	2.71	0.49	2 – 3
**Groove**	2.86	0.38	2 – 3
**Wall**	2.29	0.76	1 - 3

[illegible]

Table S9: Positive environmental samples per operation for Operator 2, grouped according to sampling time point.

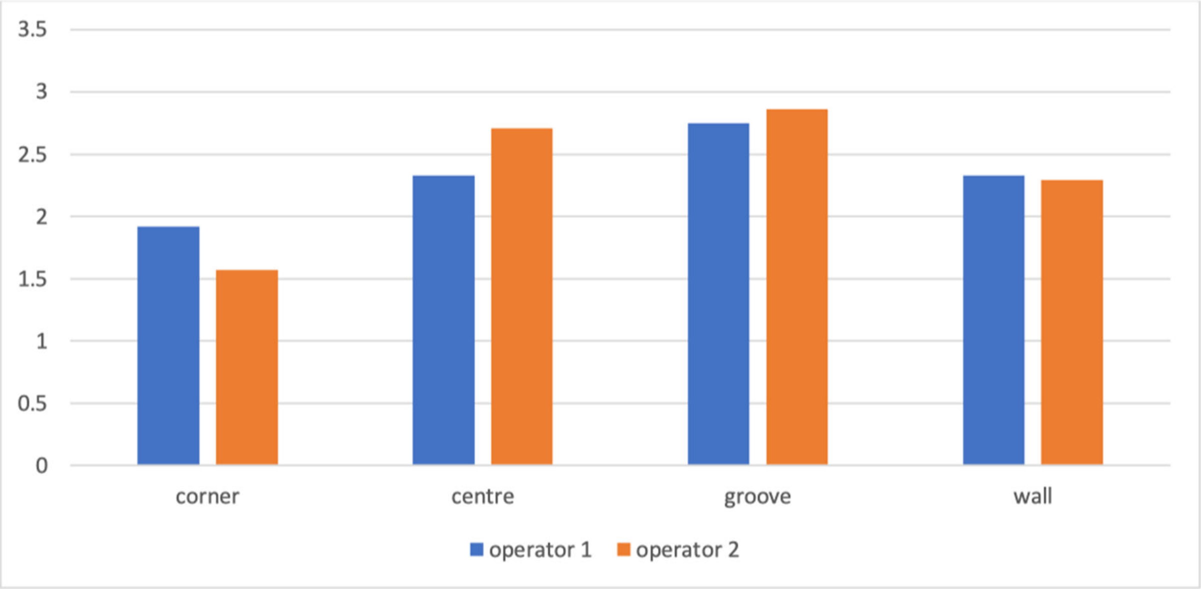

Figure S4: Column graph illustrating the relationship between the area sampled (x-axis) and contamination, expressed as the mean number of positive samples (y-axis), for each of the two operators evaluated.

	Corner	Centre	Groove	Wall
T1	67	100	100	67%
T2	100	100	100	100
T3	33	67	67	67

Table S10: Positive environmental samples per operation for Operator 1, grouped according to sampling location.

	Positive samples (all four areas):		
	Mean	Standard Deviation	Min-max
T1	3.33	0.577	3 – 4
T2	4.00	0.000	4 – 4
T3	2.33	2.080	0 - 4

Table S11: Positive environmental samples per operation for Operator 2, grouped according to sampling location.

	Positive samples (all three sampling times):		
	Mean	Standard Deviation	Min-max
Corner	2.00	1.000	1 – 3
Centre	2.67	0.577	2 – 3
Groove	2.67	0.577	2 – 3
Wall	2.33	0.577	2 - 3

Table S12: Distribution of positive environmental samples from coeliotomy procedures according to sampling location and sampling time point.

	Corner	Centre	Groove	Wall
T1	67	67	100	67
T2	56	89	89	100
T3	44	78	89	78

Table S13: Positive environmental samples per operation from coeliotomy procedures, grouped according to sampling time point.

	Positive samples (all four areas):		
	Mean	Standard Deviation	Min-max
T1	3.00	0.87	2 – 4
T2	3.33	1.00	1 – 4
T3	2.89	1.05	1 - 4

Table S14: Positive environmental samples per operation from celiotomy procedures, grouped according to sampling location.

	Positive samples (all three sampling times):		
	Mean	Standard Deviation	Min-max
Corner	2.67	0.870	1 – 3
Centre	2.33	1.000	0 – 3
Groove	2.78	0.670	1 – 3
Wall	2.44	0.527	2 – 3

Table S15: Distribution of positive environmental samples from orthopedic procedures according to sampling location and sampling time point.

	Corner	Centre	Groove	Wall
T1	43	100	100	71
T2	71	100	100	86
T3	71	57	98	57

[illegible]

Table S16: Positive environmental samples per operation from orthopedic procedures, grouped according to sampling time point.

	Positive samples (all four areas):		
	Mean	Standard Deviation	Min-max
T1	3.14	0.69	2 – 4
T2	3.57	0.53	3 – 4
T3	2.71	0.76	2 - 4

Table S17: Positive environmental samples per operation from orthopedic procedures, grouped according to sampling time point.

	Positive samples (all three sampling times):		
	Mean	Standard Deviation	Min-max
Corner	1.86	0.90	1 – 3
Centre	2.57	0.53	2 – 3
Groove	2.86	0.38	2 – 3
Wall	2.14	1.21	0 - 3

Table S18: Distribution of positive environmental samples from urogenital procedures according to sampling location and sampling time point.
